# Development of a biosensor protein bullet as a fluorescent method for fast detection of *Escherichia coli* in drinking water

**DOI:** 10.1371/journal.pone.0184277

**Published:** 2018-01-05

**Authors:** Ignacio Gutiérrez-del-Río, Laura Marín, Javier Fernández, María Álvarez San Millán, Francisco Javier Ferrero, Marta Valledor, Juan Carlos Campo, Natalia Cobián, Ignacio Méndez, Felipe Lombó

**Affiliations:** 1 Research Group BIONUC, Departamento de Biología Funcional, Área de Microbiología, University of Oviedo, Oviedo, Principality of Asturias, Spain; 2 Department of Electric, Electronic, Computer and Systems Engineering, University of Oviedo, Campus of Gijón, Gijón, Principality of Asturias, Spain; 3 HIPSITEC S.A., Oviedo, Principality of Asturias, Spain; National Central University, TAIWAN

## Abstract

Drinking water can be exposed to different biological contaminants from the source, through the pipelines, until reaching the final consumer or industry. Some of these are pathogenic bacteria and viruses which may cause important gastrointestinal or systemic diseases. The microbiological quality of drinking water relies mainly in monitoring three indicator bacteria of faecal origin, *Escherichia coli*, *Enterococcus faecalis* and *Clostridium perfringens*, which serve as early sentinels of potential health hazards for the population. Here we describe the analysis of three chimeric fluorescent protein bullets as biosensor candidates for fast detection of *E*. *coli* in drinking water. Two of the chimeric proteins (based on GFP-hadrurin and GFP-pb5 chimera proteins) failed with respect to specificity and/or sensitivity, but the GFP-colS4 chimera protein was able to carry out specific detection of *E*. *coli* in drinking water samples in a procedure encompassing about 8 min for final result and this biosensor protein was able to detect in a linear way between 20 and 10^3^ CFU of this bacterium. Below 20 CFU, the system cannot differentiate presence or absence of the target bacterium. The fluorescence in this biosensor system is provided by the GFP subunit of the chimeric protein, which, in the case of the better performing sensor bullet, GFP-colS4 chimera, is covalently bound to a flexible peptide bridge and to a bacteriocin binding specifically to *E*. *coli* cells. Once bound to the target bacteria, the excitation step with 395 nm LED light causes emission of fluorescence from the GFP domain, which is amplified in a photomultiplier tube, and finally this signal is converted into an output voltage which can be associated with a CFU value and these data distributed along mobile phone networks, for example. This method, and the portable fluorimeter which has been developed for it, may contribute to reduce the analysis time for detecting *E*. *coli* presence in drinking water.

## Introduction

Water is an essential resource for all life forms and its quality is under constant challenge at water treatment plants and along transportation pipes towards consumer´s tap. Throughout this journey, and especially in its source, water is exposed to an increasing number of infectious, chemical and radioactive pollutants that can be ingested by humans, causing serious health problems [1].

Therefore, drinking water may, in certain cases, be the cause of disease in humans. The reason is that water can act as a vector of a large number of environmental toxic compounds, such as toxins of microbial (cyanobacterial hepatic toxins) or mineral origin (mercury, radioactive compounds). These toxins may be derived from human activities, as poisons of industrial origin (dioxins, organotin compounds). Furthermore, drinking water can also be the gateway to our organism for different pathogens, such as parasites, fungi, bacteria, or viruses; by means of faecal contamination or improper water treatment [2]. Countries such as Norway or the USA have described massive outbreaks of intestinal parasites such as *Giardia*, *Cryptosporidium* and *Entamoeba*, due to problems along the networks of water supply [3], [4]. In Canada, *Toxoplasma* infections cause neurological damage in newborns whose annual treatment costs around 200 million € [5]. Bacteria with high mortality rates like *Leptospira* o *Legionella* are transmitted by water distribution networks with poor maintenance, affecting hundreds of people [6,7]. In tropical countries, cholera cases, transmitted by consumption of contaminated water, may increase to hundreds of thousands during outbreaks [8]. In the case of viruses, the most common one transmitted by contaminated water is hepatitis A virus, with about 1.5 million people affected yearly [9]. Therefore, access to clean drinking water is essential, leading to significant health benefits [10].

As a consequence, contamination of drinking water with bacteria and other pathogens remains a major cause of health problems in industrialized and developing countries [11]. The WHO/UNICEF Joint Monitoring Program for Water Supply and Sanitation estimates that 663 million people lack access to safe drinking water sources [12], this being a leading cause of death worldwide (only after respiratory infections and AIDS) with 3.4 million deaths annually [13–15]. It is estimated that 4% of annual deaths and 5.7% of annual morbidity are caused by waterborne diseases, and some areas are particularly vulnerable to waterborne diseases, due to their underdeveloped infrastructure and lack of enough water treatment plants. However, the distribution of drinking water and wastewater in high-income areas also require monitoring of microorganisms and contaminants [16]. In fact, in countries like USA, it has been estimated that 7.1 million people contract waterborne infections each year, of whom 12,000 die annually [10].

To ensure its safety, drinking water must meet diverse requirements, such as absence of pathogens and certain chemicals, as well as being tasteless, colourless and odourless [17]. However, it is impossible to sample every hour at each point along distribution pipes, and it is not feasible to detect all possible pathogens or toxins in these assays. So most countries have established a consensus protocol by which, once or few times per day, drinking water is tested in search for the presence of the main chemical toxins (heavy metals, pesticides, etc.) and a few microbial indicators. The absence of these bacterial indicators is considered to be a proof of good microbial quality, as they are sentinel species of faecal origin. The most commonly used bacterial water indicators are *Clostridium perfringens*, *Enterococcus faecalis* and *Escherichia coli*, which must be absent (for example, in 100 mL water samples) in order to assure that any drinking, recreational or farming water is safe for humans [18–21]. *E*. *faecalis* is mainly used as an additional microbiological indicator when an *E*. *coli* single analysis is not suitable because of the suspicion of its possible multiplication in tropical waters [22]. On the other hand, *C*. *perfringens* is barely used as a unique microbiological indicator because of its extremely long half-life [22]. For all of these reasons, *Escherichia coli* was chosen in 1890 as the main reference indicator to assess the microbiological quality of drinking water, as it meets all the conditions for a good sentinel bacterial: it is present in all mammals faeces in large amounts, it hardly multiplies outside the host, and it is detectable by sensitive methods that require simple services at a bacteriological laboratory [23,24].

A common method considered to be the gold-standard for detection of these indicator bacteria is its growth on selective agar media for enterobacteria, which contains inhibitors for preventing growth of other unwanted bacteria. However, these traditional culture methods used for quantification of indicator bacteria require days to obtain results. Therefore, there is an urgent need for the development of a cheap, fast and reliable method to detect *E*. *coli* in drinking water [11,25]. Water safety would increase with the development of faster and portable methods for *E*. *coli* detection, allowing multiple sampling sites and many inexpensive tests per day. These real time biosensors would therefore diminish the lapse of alarm time between any eventual contamination along distribution pipes and the health authority’s response. Current developed methods for fast detection include DNA hybridization, PCR (and qRT-PCR for quantification), immunoassays, immunomagnetic separation, lateral flow tests, incubation micro-chambers as VITEK, labelled nanoparticles, NIRS (near infrared spectroscopy), DEP-FFF (dielectrophoretic field-flow fractionation) and β-D-glucuronidase assays [26–34]. But these current technologies are not inexpensive neither in equipment (as NIRS) nor in reagents (like labelled antibodies), and usually require an intense manipulation of water samples and laboratory equipment (as DNA-hybridization), which impairs its portability as well as the feasibility of testing every few minutes. Another low-cost and easy to handle technologies have been proposed for the detection of pathogenic bacteria, including colorimetric, electroluminescence, immunomagnetic detection and electrochemical methods. Moreover, recent advances in nanotechnology have enabled the development of new technologies based on nanoparticles, nanorods, nanosheets and 3D-nanostructures for rapid and sensitive detection of pathogens [35], but most of these methods are still very expensive for commercial use and require sophisticated instrumentation [16].

However, in recent years an intensive research has been conducted to simplify these technologies, generating portable biosensors with immediate *in situ* results. These detection biosensor assays must also be both sensitive (able to detect low concentrations of the target agents without the interference from other water particles) and specific (for example, for *E*. *coli* and not for other enterobacteria) [36,37]. In this work, we describe the development of a portable biosensor system (578 g) for fast (8 min) and sensitive (10 CFU/L) detection of *E*. *coli* in drinking water samples, with a linear range for over 20 to 10^3^ CFU. Three different approaches have been made, in order to develop these chimera proteins as specific and sensitive biosensors for *E*. *coli* in drinking water, however, only one of them, containing a GFP domain linked to a colicin S4 bacteriocin domain, has performed the required binding capabilities to the bacterial cells. The low cost of this equipment and its automation capabilities make this biosensor a real possibility to monitor multiple places along water distribution, eventually sending this information in real time to central control offices, for example by using a mobile phone network. The main objective of the method described in this study is to control the quality of water in a cheap way and in multiple locations throughout the distribution system as well as to rapidly process samples and to share real-time test results.

## Material and methods

### Bacterial strains, plasmids and culture conditions

*E*. *coli* TOP10 F’ (Invitrogen, a strain with wt genes coding for for Fhu and OmpW), and cloning vectors pUC57 (GenScript) and pIAGO [38] were used for routine sub-cloning while *Streptomyces albus* was used as host for heterologous protein expression. *E*. *coli* clones were incubated at 37°C and 250 rpm in TSB (Tryptic Soy Broth, Merck) or LB (Luria-Bertani Broth, Merck) for growing in liquid medium, and TSB containing agar was used for growing on solid medium. Appropriate concentrations of antibiotics were added for plasmid selection: 100 μg/mL ampicillin for both vectors. *S*. *albus* was sporulated on Bennet medium [39] at 30°C and grown in liquid YEME medium for protoplasts preparation [40]. Transforming colonies were cultured at 30°C on R5 solid medium, sporulated on Bennet medium and subcultured in R5A liquid medium [41] for protein expression. Spores were kept in glycerol 25% at -20°C. Media were supplemented with thiostrepton at 5 or 50 μg/mL (liquid or solid cultures respectively). *Enterobacter cloacae* (strain CECT 194T) and *Salmonella enterica* var. *arizonae* (strain CECT 4395) were used as negative controls, and have been grown in TSB medium as well.

### DNA manipulation

Plasmid DNA extraction, restriction endonuclease digestion, DNA ligation, transformation and other DNA procedures were performed according to standard techniques for *E*. *coli* [40]. Preparation of *S*. *albus* protoplasts, transformation and selection of positive transforming colonies were carried out as described [39]. Restriction enzymes, buffers and T4 DNA ligase were purchased from EURx. The three chimera proteins and the ermE*-rbs promoter were obtained by gen synthesis (GenScript).

pb5 phage protein (accession number AAU05292.1) was chosen as a sensor bullet for the detection of *E*. *coli*. The synthetic gene coding for Green Fluorescent Protein (GFP) (accession number AAA27722) was fused to the synthetic gene coding for pb5 protein (640 amino acids). At the 5’ end of GFP gene, a polyhistidine-tag coding sequence was fused in order to facilitate further protein purification. A synthetic DNA coding for a flexible peptide linker with the structure of a random coil was designed between GFP and pb5 domains, (ELFLRSLQDKYSNLHFHVPTPLDPHTHVKQIDKYDLSNLHFHLPGHKAPDNKGPYTPKKGDPPKGLDTGKPTGHNQRGHYPLLNNPEATNAGKYEYWPSH). This encoded flexible linker contains neither α-helixes nor β-sheets (tested with ExPASy tools: CFSSP, HMMTOP and TMHMM), and therefore facilitates anchoring of pb5 bullet domain to *E*. *coli* outer cell structures, without hindering GFP function. The gene coding for the biosensor chimera protein GFP-pb5 (accession number KY404234) was synthesized and codon-optimized for *Streptomyces* expression, adding extra restriction enzyme sites for cloning purposes.

Hadrurin peptide (accession number NDB21_HADAZ), from a scorpion venom, was chosen as another sensor bullet protein for the detection of *E*. *coli*. A polyhistidine-tag sequence was designed, fused to GFP (accession number AAA27722) and this to hadrurin. The same linker between GFP and hadrurin was used, and a synthetic gene coding for a biosensor chimera GFP-hadrurin (accession number KY404233) was also synthesized and codon-optimized for its expression in *Streptomyces*, adding extra restriction enzyme sites for cloning purposes as it has been said before.

Colicin S4 bacteriocin protein (accession number CAB46008) was chosen as the third sensor bullet protein for the detection of *E*. *coli*. For this third biosensor chimera protein, it was also fused to GFP (accession number AAA27722) and to a polyhistidine-tag sequence. The same linker was used as well. The synthetic gene coding for the biosensor chimera GFP-S4 (accession number LT548289) was synthesized and codon-optimized, adding extra restriction enzyme sites. All three synthetic genes contained a rbs for use in *Streptomyces*.

Finally, in order to improve protein expression, the strong ermE* promoter (accession number M11200, P_ermE*_) was used as well. This promoter allows a strong constitutive expression of the protein in *Streptomyces* genus.

### Construction of three expression plasmids

pUC57 vector, containing GFP-pb5 synthetic gene construction (2,967 nt insert, pJFF13-GFT5) was digested *Xba*I-*Hind*III and the resulting fragment was subcloned into the bifunctional multicopy vector pIAGO, giving rise to plasmid pJFF*-BS2-NP. The ermE* promoter present in pIAGO vector ensures the proper translation of the cloned gene, generating the final construction named pJFF1*-BS2-NP. This plasmid was sequenced to confirm its identity and transformed into *S*. *albus* protoplasts.

Also, pUC57 vector containing GFP-hadrurin synthetic gene construct (1,173 nt, pMAS12-GFHD) was digested *Xba*I/*Hind*III and the resulting fragment was subcloned into pIAGO, giving rise to plasmid pMAS40*-BS2-NP. This plasmid was sequenced to confirm its identity, and transformed into *S*. *albus* protoplasts.

Finally, pUC57 vector containing GFP-colicin synthetic gene construct (2,757 nt, pMAS11-HPS4) was digested *Xba*I/*Hind*III and the resulting fragment was subcloned into pIAGO, giving rise to plasmid pLMF*-BS2-NP. This plasmid was sequenced to confirm its identity and transformed into *S*. *albus* protoplasts.

### Protein extraction and purification

Spores of *S*. *albus* (10^7^ spores/mL) harboring pJFF1*-BS2-NP, pMAS40*-BS2-NP and pLMF*-BS2-NP were grown in 25 mL of TSB medium as preinoculum. Thiostrepton was added as selective antibiotic and the culture was grown overnight at 30°C and 250 rpm.

Each one of the three preinocula was used for inoculating two 2500 mL Erlenmeyer flasks, each one containing 250 mL R5A culture medium supplemented with glycine (0.5% final concentration) and thiostrepton. These production cultures were grown for 2 days at 30°C and 250 rpm. After centrifugation, cell pellets were washed twice with 10.3% sucrose and then resuspended in PBS buffer with 2 mg/mL lisozyme plus EDTA-free protease inhibitors mix (Thermo Scientific) and incubated for 60 min at 4°C. Protoplasts formed after this lysis step were broken by sonication (4°C, 4 pulses of 30 s, 1 min stop between pulses). After sonication, cell solutions were centrifuged at 15,557 rcf for 10 min at 4°C in order to get rid of cellular debris.

Supernatant was then used for protein purification by FPLC in two steps. First, a 5 mL HisTrap column (GE Healthcare) was used for purification of His-tag GFP-linker-bullet biosensor chimera proteins. Elution buffer A was 20 mM imidazole, 0.2 M sodium phosphate buffer pH 7.1 and 0.5 M NaCl; elution buffer B was 500 mM imidazole, 0.2 M sodium phosphate buffer pH 7.1 and 0.5 M NaCl. A gradient was applied between buffers A and B with a 2.5 mL/min flow rate during 40 min. Fluorescent fractions from this first column purification step were combined and concentrated in order to change the buffer to PBS buffer with EDTA-free protease inhibitors (Thermo Scientific). These protein concentration steps were carried out with Pall Macrosep Advance centrifugal devices 10K MWCO (for GFP-hadrurin biosensor chimera) or 30K MWCO (for GFP-pb5 and GFP-colicin S4 biosensor chimeras). Concentrated protein solutions (1 mL final volume) were then further purified with Superdex 200 10/300 GL column (GE Healthcare), as a size exclusion second purification. Purified proteins were quantified by using Bradford and stored at 4°C in PBS with protease inhibitors.

### Electronic measurement system

**Fig 1:**
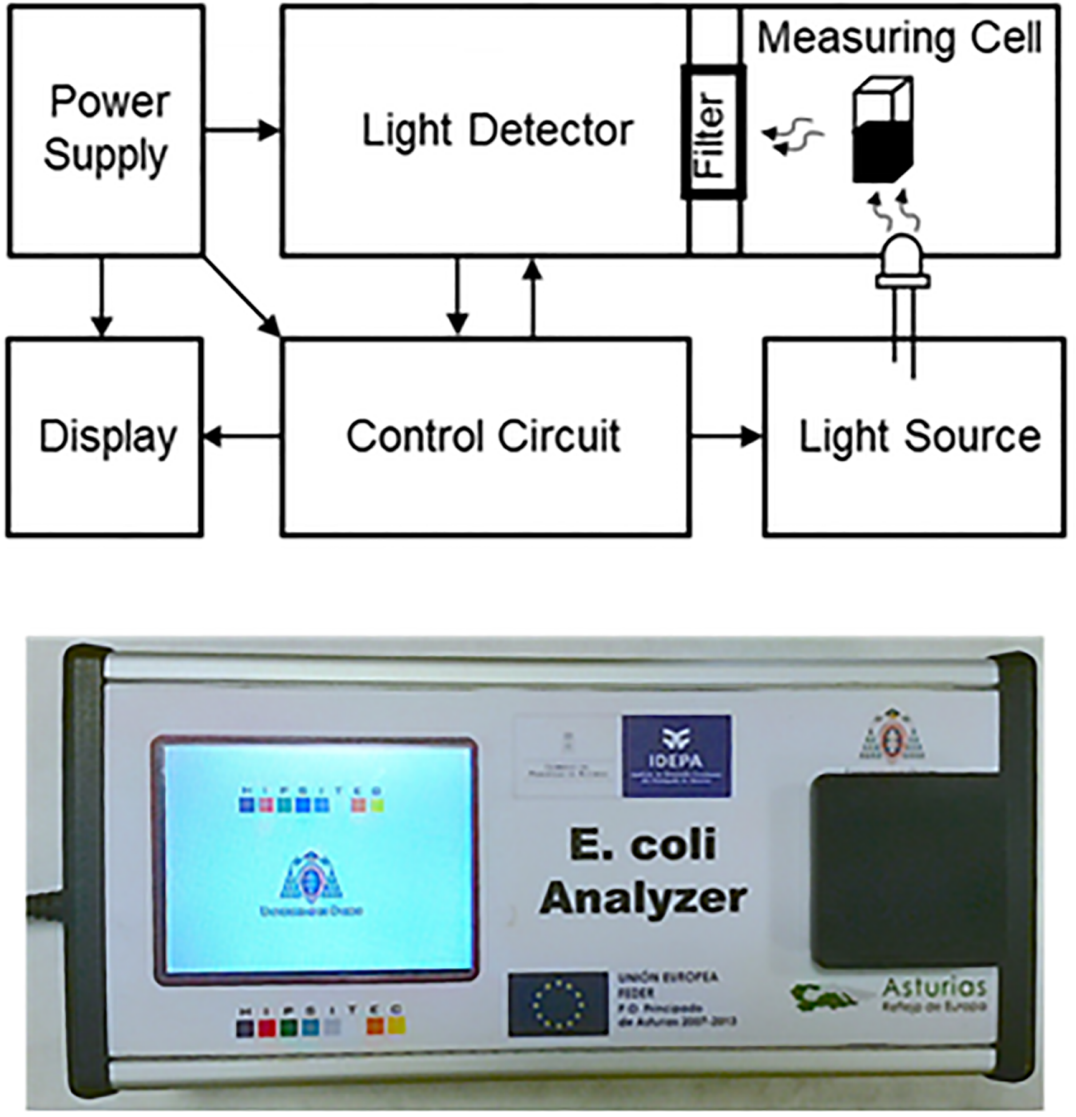
Functional blocks of the measurement system at the portable *E*. *coli* Analyzer device.

A portable measurement device has been developed for detection of *E*. *coli* cells, once these cells may have been labelled with the corresponding fluorescent biosensor chimera protein. The construction of this *E*. *coli* Analyzer device has been already described [42]. The rationale of the measurement is based on the detection of the fluorescence emitted by the GFP protein domain immobilized in *E*. *coli* cells, via the linker-bound bullet protein domain of each designed biosensor chimera. The system comprises a light source which excites the fluorescent chimera protein (Fig 1). Then, the light emitted by this biosensor chimera protein (bound to *E*. *coli*) is filtered before reaching the photomultiplier optical detector [43]. The light signal is finally converted into an electric signal output (Fig 1). The power supply allows the instrument to be charged from the power grid or by rechargeable batteries for applications on-site, allowing a convenient portability of the device.

The optical subsystem in the device consists of the light source, the measuring cell (for getting 2 mL cuvettes) and the light detector. This design was carried out on the basis of the excitation and emission spectra of GFP protein, where the excitation peak of greatest intensity is at 395 nm. Therefore, this wavelength is the corresponding to the peak of the light source emission (LED diode) located in the portable device [42,44].

Exposed to this LED excitation source, the GFP domains of the biosensor chimera proteins emit a fluorescence whose peak is at 509 nm. Therefore, the light detector incorporated in the *E*. *coli* Analyzer portable device is highly sensitive to this wavelength.

Finally, the device microchip microcontroller reads the value of fluorescence intensity coming from the photomultiplier and it converts this intensity to an output mV (Fig 1). It also adjusts the value of the gain of the PMT (via touch display), manages the process of calibration, etc.

The calibration process is performed before each sample measurement. This step is critical in order to obtain good results. For this, a sample of PBS buffer containing the corresponding *E*. *coli* dilution (blank sample) is placed into the measuring cell and the output voltage read under these condition is stored. If the system were ideal this voltage should be zero volts, but the photomultiplier generates a certain offset voltage. Then, the real water sample to be analyzed is placed in the cuvette, and the control system reads the voltage value obtained and subtracts the offset voltage. Finally, interpolating this value in the calibration curve, the number of CFUs is obtained and it is shown in the display.

### Detection assay

A single colony of *E*. *coli* Top10 was inoculated in 5 mL of LB medium and incubated at 37°C until reaching an OD of 0.8 measured at 600 nm with a Biophotometer (Eppendorf). Decimal serial dilutions from this culture were done in PBS buffer (from 3 to 3x10^5^ CFUs/mL).

2 mL of each purified biosensor chimera protein solution were incubated with 100 μL of the corresponding *E*. *coli* dilution for 5 min (room temperature, 100 rpm vortex). This mixture (2100 μL) was then filtrated using a 0.2 μm cellulose acetate filter cartridge (Whatman). In order to eliminate the unbound biosensor chimera protein on the filter membrane, 2 mL of extra PBS buffer was used as a wash step.

At this point, *E*. *coli* cells (with and without bound biosensor chimera proteins) were eluted with 2 mL of PBS buffer, using the same filter, but in the reverse position; this step allowed to recover all cells present on the filter. Fluorescence was measured in 2 mL fluorimeter cuvettes (VWR) using this 2 mL recovered solution, and this measurement was carried out with the developed *E*. *coli* Analyzer device. Blank solution for these measurements was PBS buffer with the corresponding *E*. *coli* dilution and protease inhibitors, these PBS blank samples were measured before each experimental sample in order to eliminate putative background interferences. This device registered for each binding assay a mV value which corresponds to the equivalent fluorescence emitted by the biosensor chimera protein bound to the *E*. *coli* cells present in the experiment, and this mV value is automatically referred to a CFUs measure which was established by using a gold standard test. This test was carried out by plating out each dilution on EMB agar plates and incubating them overnight at 37°C [45].

### Confocal microscopy

Images were collected with Leica TCS-SP2-AOBS and Leica TCS-SP8X confocal microscopies (Servicios Científico-Técnicos, Universidad de Oviedo) using a 63x/1.40 Oil objective. GFP was excited using a 488 nm argon/krypton ion laser and a white laser, and fluorescence emission was detected at 502–556 nm. Leica Confocal Software (LCS) version 2.61 Build 1537 and Leica Application Suite X (LASX) were used for data acquisition (Leica Microsystems Heidelberg GmbH (Germany)). *S*. *albus* transformation clones for pJFF1*-BS2-NP, pMAS40*-BS2-NP and pLMF*-BS2-NP plasmids were cultivated in R5A liquid medium, and these mycelia were analyzed by confocal microscopy in order to detect production of the corresponding fluorescent biosensor chimera protein. *E*. *coli* samples used for binding assays of each chimera fluorescent protein were analyzed under confocal microscope as well, following the same protocol that has been described in the “Detection assay” section.

Also, in order to determine if binding of the fluorescent biosensor chimera protein to *E*. *coli* cells surface was stable in each of the three cases, the recovered *E*. *coli* solution (after chimera binding) was filtered again before a second round of confocal microscopy studies.

## Results

Three proteins with binding capabilities to *E*. *coli* have been chosen and analyzed as biosensor alternatives for this system. The first option was hadrurin, a small protein (41 amino acids) from the venom cocktail of the Mexican scorpion *Hadrurus aztecus* [46]. The second biosensor protein chosen was the tail protein pb5 from T5 phage, which is the one interacting with the receptor protein at the outer membrane of *E*. *coli* [47]. Finally, the third alternative was colicin S4, a bacteriocin recognizing *E*. *coli* cells, which was actually the only one providing good performance in the detection tests [48,49].

### GFP-Hadrurin biosensor chimera protein

Hadrurin is an antimicrobial peptide (AMP) isolated from the venom of *Hadrurus aztecus* scorpion. AMPs have a broad spectrum of target bacteria and induce cell death in a short contact time [50]. This antibacterial peptide is composed of 41 amino acids with a molecular mass of 4.4 kDa. Its antimicrobial effect is present at low concentrations, in a micromolar range, inhibiting the growth of a wide range of bacteria, including *E*. *coli*. A specific receptor for the action of hadrurin has not been described, and the most likely mechanism of action would be by destabilization of the cytoplasmic membrane activity due to formation of transient pores [46,50].

**Fig 2:**
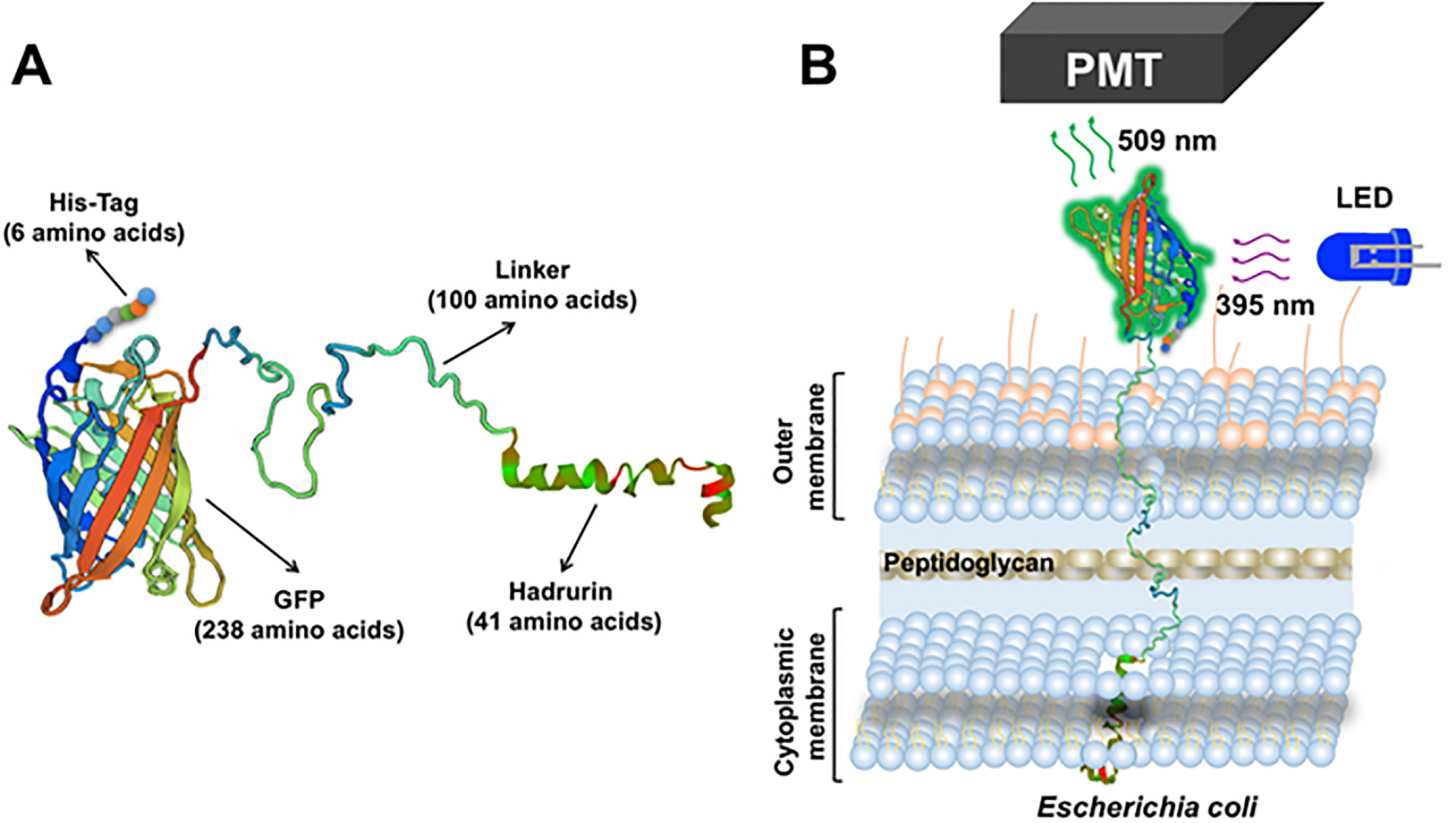
A: Hypothetical spatial structure of the fluorescent biosensor chimera protein GFP-hadrurin. B: Diagram showing the attachement process of the fluorescent biosensor chimera protein to the membrane of *E*. *coli*. PMT: photomultiplier (part of the *E*. *coli* Analyzer device in charge of detecting and amplifying the fluorescence signal produced by the GFP domain of each biosensor chimera protein, after receiving the 395 nm excitation light from the LED source).

The hadrurin peptide was selected as a first candidate for testing it as a sensor protein targeting *E*. *coli* cells in our method (Fig 2). Therefore, *S*. *albus*-pMAS40*-BS2-NP spores were cultivated in 250 mL R5A production medium in order to get cell mass to isolate GFP-hadrurin biosensor chimera protein (386 amino acids, Fig 2A). After FPLC purification steps, a concentration of 152 μg/mL sensor bullet was obtained (measured by Bradford). 2 mL of GFP-hadrurin protein were used for binding assays to 100 μL of each *E*. *coli* dilution (see Materials and methods section).

In order to visualize binding of the GFP-hadrurin biosensor chimera protein to cells, the fluorescence was measured with the *E*. *coli* Analyzer fluorimeter (see Materials and methods section). However, these experiments showed low fluorescence signals.

**Fig 3:**
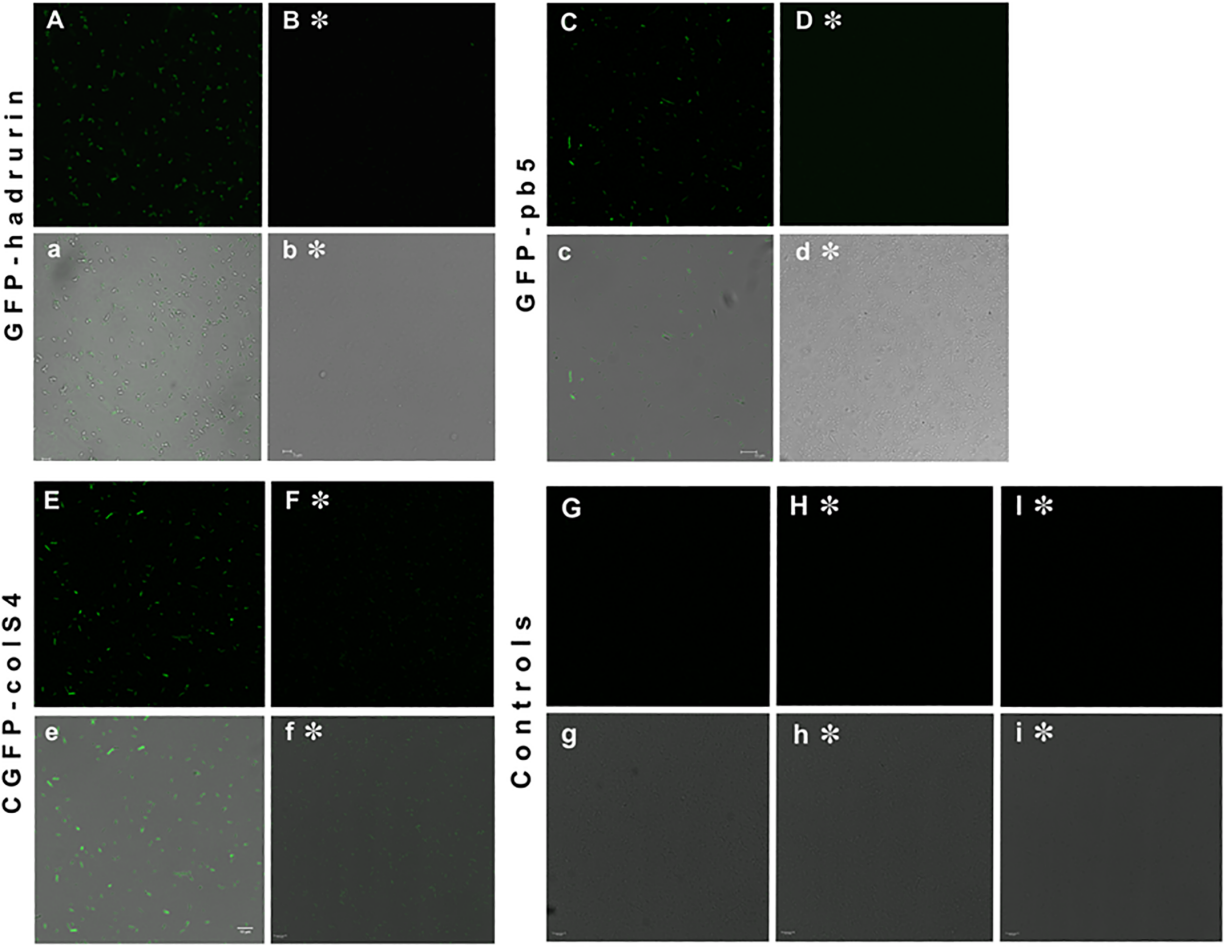
Confocal microscopy images of *E*. *coli* (A–G, a–g), *Salmonella enterica* (H, h) and *Enterobacter cloacae* (I, i) fluorescent labeling tests. Each group of 4 images correspond to a different chimeric biosensor fluorescent protein. Images from “A” to “I” correspond to confocal microscopy, showing the fluorescent labelled cells, and images from “a” to “i” are the same confocal images but fused with an optical image generating a single photo (merged snapshot for testing that fluorescent spots actually correspond to labelled cells, and not to background fluorescence). The symbol “✽” means that the sample has been filtered in order to get rid of unbound chimera protein. A-B, images of *E*. *coli* labelled with GFP-hadrurin fluorescent chimera protein, where labeling is lost after a filtering step. C-D, images of *E*. *coli* labelled with GFP-pb5 fluorescent chimera protein, where labeling is also lost after a filtering step. Only the fluorescent biosensor chimera protein GFP-colS4 (E,F) is able to maintain its strong binding to the *E*. *coli* surface after the filtration step. Also, the other two bacterial species (negative controls) are not labelled at all (H, I). Images G and g are negative controls for *E*. *coli*, where no fluorescent biosensor chimera protein has been added, as a method to test that this bacterial cells do not show autofluorescence under these conditions.

We decided to analyze the same samples under confocal microscope, with and without the filtering step used to get rid of unbound biosensor chimera protein after mixing the protein with the *E*. *coli* dilutions. Confocal images showed that GFP-hadrurin labeling of cells was very low once the labeled cells were filtrated (Fig 3). As this GFP-hadrurin low labeling resulted in low signals, no further experiments were carried out with this chimera.

### GFP-pb5 biosensor chimera protein

The infection process of Gram-negative bacteria by a bacteriophage begins with a highly specific mechanism of interaction in which the receptor binding proteins (RBP) of the phage bind particular structures exposed on the surface of the bacterium. The RBP pb5 (67.8 kDa) from phage T5 is located at the tip of the central tail fibers, near the baseplate, and ensures binding of phage T5 to its receptor, placed on the outer membrane of *E*. *coli* cells. This receptor is the outer membrane iron-ferrichrome transporter FhuA, which mediates the transport of iron, as it is usually present in very low concentrations in the environment [47].

**Fig 4:**
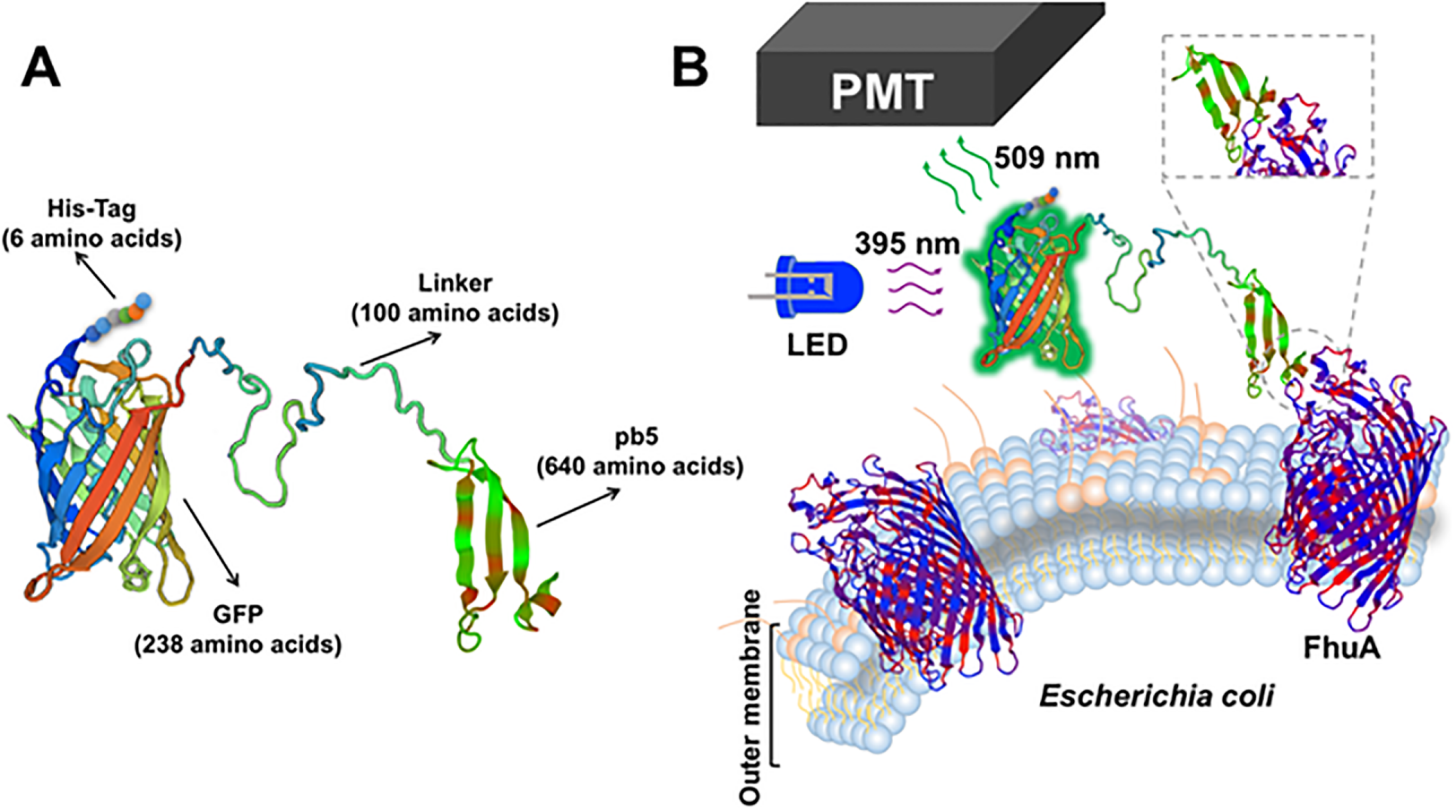
A: Hypothetical spatial structure of the fluorescent biosensor chimera protein GFP-pb5. B: Diagram showing the attachement process of the chimera GFP-pb5 to the FhuA transporter of *E*. *coli*. PMT: photomultiplier (part of the *E*. *coli* Analyzer device in charge of detecting and amplifying the fluorescence signal produced by the GFP domain of each biosensor chimera protein, after receiving the 395 nm excitation light from the LED source).

As another approach in order to develop a biosensor chimera protein for detection of *E*. *coli* in drinking water samples, a second synthetic gene was designed, in which the GFP domain was linked to the already described pb5 RBP from phage T5 (Fig 4).

This pb5, structurally, in comparison with other RBPs, it shows a high content of β-sheet and a low helical content [51]. The crystal structure of its receptor on the bacterial outer membrane, FhuA, comprises a 22 β-strands barrel that is inserted in the outer membrane of the bacteria and contains a plug which fills the lumen of the barrel. This plug is a globular N-terminal domain which penetrates the barrel from the periplasm side and closes the pore of the barrel tightly [51,52]. The plug has four-stranded short β-sheet and four short helices that are connected both to the barrel and to hydrophilic loops facing the external medium [51]. Pb5 binds to two large external loops located in the FhuA barrel [53–56].

The *in vitro* interaction between purified pb5 and FhuA generates a highly stable stoichiometric complex, which possesses the same stability as in the case of the complete phage [53–55,57]. The fact that a purified soluble pb5 protein is able to bind its receptor forming a stable complex is not obvious, because the binding proteins of the phage tail undergo several conformational changes when binding its bacterial receptor, which are transmitted to neighbouring tail proteins, initiating a series of conformational changes that convert phage tail into an appropriate structure to be crossed by DNA. Taking into account this mutual interdependence in relation to conformational changes of proteins in the phage tail, one could think that in its soluble form (free) pb5 wouldn’t be able to have an optimal conformation to facilitate the binding. However, *in vitro* studies have shown that pb5 conformation is the same as free protein and as part of phage tail [51,56,58].

Based on these data, we decided to use this GFP-pb5 biosensor chimera protein as a second candidate for specific fluorescent detection of *E*. *coli* in drinking water samples (Fig 4). To carry this out, *S*. *albus*-pJFF1*-BS2-NP spores were cultivated in 250 mL R5A production medium in order to get cell mass to isolate GFP-pb5 biosensor chimera protein (984 amino acids, Fig 4A). After FPLC purification steps, a concentration of 332 μg/mL biosensor chimera protein was obtained (Bradford quantification). 2 mL of GFP-pb5 biosensor chimera protein were used for binding assays to 100 μL of each *E*. *coli* dilution. protein is a highly unstable protein that tends to aggregate in concentrations above 500 μg/mL.

In order to visualize binding of the GFP-pb5 biosensor chimera protein to cells, the fluorescence was measured using the *E*. *coli* Analyzer fluorimeter, but these analyses rendered very low fluorescence signals.

Further analyses of these same samples were carried out under confocal microscope. Confocal images showed a better GFP-pb5 labeling of cells only in samples where no filtering step was applied. This indicated that probably, GFP-pb5 chimera protein binding to *E*. *coli* cells was labile, and that most bound GFP-pb5 was washed away during the filtration step used to get rid of unbound chimera protein (Fig 3C and 3D). Therefore, we decided to stop further experiments with GFP-pb5.

### GFP-colS4 biosensor chimera protein

Colicins have been extensively studied since its discovery in 1925. These plasmid-encoded toxic proteins are synthesized by about 50% of *E*. *coli* strains and their function is to specifically eliminate other *E*. *coli* competitive strains, under certain stress conditions [48]. The most common toxicity mechanism for colicins is the formation of a pore in the cytoplasmic membrane, followed by an interruption of the electrochemical gradient [59].

**Fig 5:**
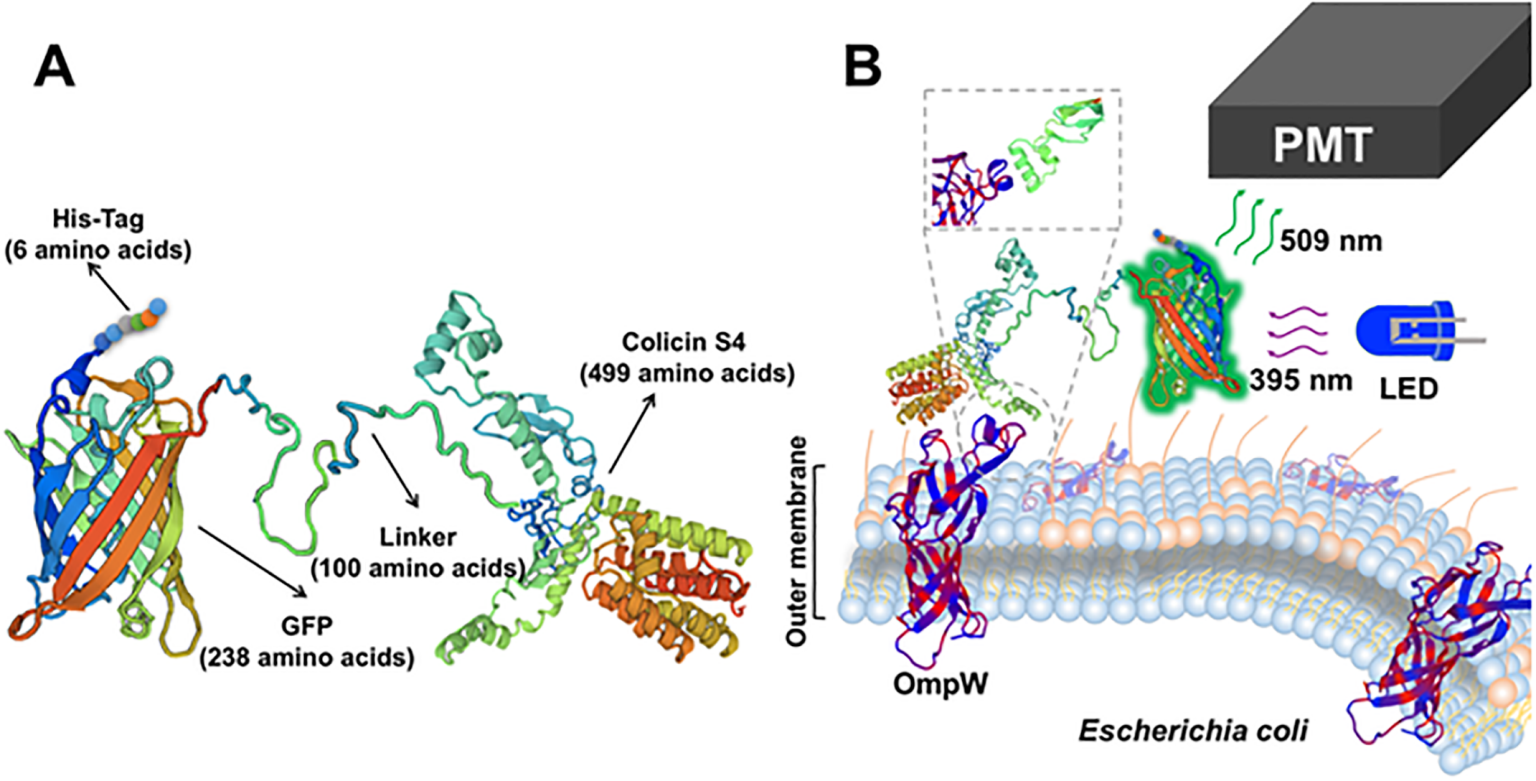
A: Hypothetical spatial structure of the fluorescent biosensor chimera protein GFP-colS4. B: Diagram showing the attachement process of the fluorescent biosensor chimera protein to the OmpW receptor of *E*. *coli*. PMT: photomultiplier (part of the *E*. *coli* Analyzer device in charge of detecting and amplifying the fluorescence signal produced by the GFP domain of each biosensor chimera protein, after receiving the 395 nm excitation light from the LED source).

Based on this knowledge, colicin S4 was chosen as a third alternative for construction of a new biosensor chimera protein. The sequence of this colicin shows a characteristic mosaic-like structure consisting of three domains: a N-terminal domain responsible for translocation through the outer membrane by interacting with the Tol proteins system; a central domain that mediates the binding to its OmpW receptor located in the outer membrane; and a C-terminal domain responsible for the pore formation. This narrow host range is determined by a highly specific binding of the colicin S4 into *E*. *coli* sensitive cells [48,49,60] (Fig 5).

The initial contact between colicin S4 and *E*. *coli* cell is established between the conserved receptor binding domain and the OmpW specific receptor in the bacterial outer membrane which is a member of a large family of small β-barrel proteins. After this receptor recognition, the translocation domain can penetrate through the pores of the OmpW receptor across the outer membrane, towards the periplasm. Once in the periplasm, the colicin acts on the plasma membrane, generating pores [61].

Colicin S4 is the only colicin containing two identical receptor binding domains, both recognizing *E*. *coli* OmpW protein, but the binding of one of them is enough for triggering an effect on the *E*. *coli* cell [48]. OmpW is structured in eight transmembrane β-strands with extended loops exposed to the environment [62]. The first helix from both colicin S4 receptor binding domains (R_1_α1 and R_2_α1) are able to interact with the Asp^116^, His^117^ and Glu^120^ amino acids of OmpW, which are freely exposed to the extracellular space [48,63].

In order to test the new GFP-colS4 chimera protein as a new candidate for florescent labeling of *E*. *coli* cells (Fig 5), *S*. *albus*-pLMF*-BS2-NP spores were cultivated in 250 mL R5A production medium in order to get cell mass to isolate GFP-colicin S4 biosensor chimera protein (843 amino acids, Fig 5A). After FPLC purification steps, a concentration of 537 μg/mL biosensor protein was obtained (measured by Bradford). 2 mL of GFP-colicin S4 protein were used for binding assays to 100 μL of each *E*. *coli* dilution (see Materials and methods section).

**Fig 6:**
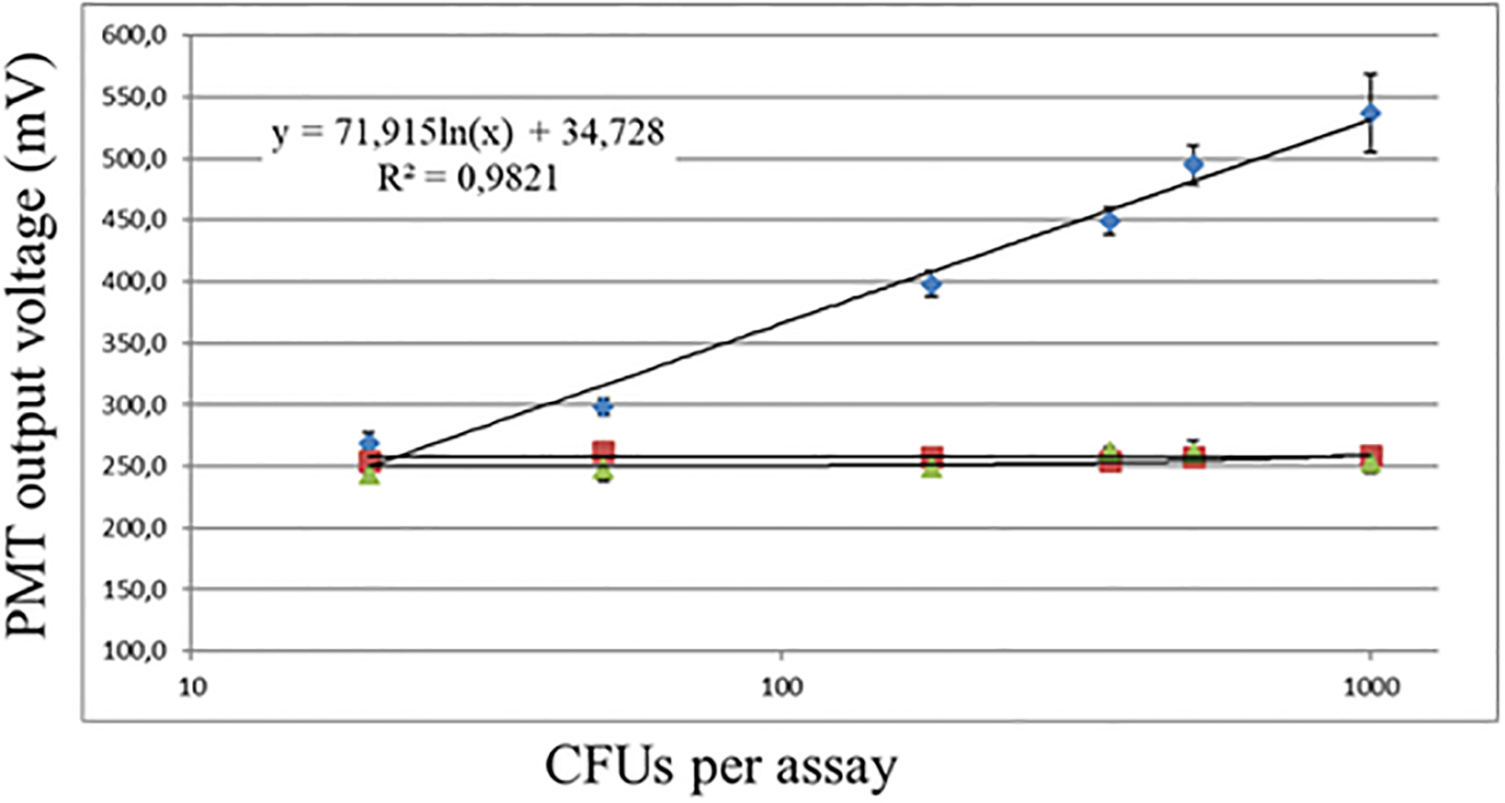
Sensitivity curve using GFP-colS4 as biosensor fluorescent chimera protein for *E*.*coli* (blue dots) at diverse CFU concentrations, from 20 to 10^3^ CFU, which is the detected linear range. Dots represent the average values and the standard error from three different detection experiments at each condition. Over 10^3^ CFU the method shows saturation. Error bars for each triplicate experiment are shown. Data for *S*. *enterica* var. *arizonae* (green dots) and *E*. *cloacae* (red dots) are also shown.

In order to visualize binding of the GFP-colicin S4 biosensor chimera protein to *E*. *coli* cells, the fluorescence was measured with the *E*. *coli* Analyzer fluorimeter, getting good fluorescence signal values (obtained as mV output), which were used for generating the sensitivity curve (Fig 6). This curve shows that in binding experiments with more than 3x10^3^ *E*. *coli* CFU, the system gets saturated, and then there is not a further increase in the generated mV output signal. Also, with *E*. *coli* CFU below 20, the signal is also no longer proportional to the number of *E*. *coli* CFU, as signal intensities below these 20 CFU are three times of standard deviation lower than the mean intensity from background signal (S1 Table). Therefore, the lineal signal range obtained with these experiments is between 20 and 3x10^3^ *E*. *coli* CFU. (Fig 6).

We decided to analyze the same GFP-colicin S4 samples under confocal microscope, with and without the filtering step (which is used for getting rid of the unbound chimera protein). Confocal images showed that GFP-colicin S4 labelled *E*. *coli* cells perfectly, even after the filtration step, a procedure which causes unbinding in the cases of GFP-hadrurin and GFP-pb5 experiments (Fig 3E and 3F).

Moreover, in order to test the specificity of this method for *E*. *coli*, the same labeling experiments were carried out with dilutions of *Enterobacter cloacae* and *Salmonella enterica* var. *arizonae* as negative controls. These experiments showed that GFP-ColS4 biosensor chimera protein was not recognizing these other two species (Figs 6 and 3H and 3I), validating the fact that this specificity of the GFP-colS4 chimera towards *E*. *coli* is not due to an unspecific binding through the GFP nor the central linker peptide domains, as these ones are also present in the two other chimeric proteins, which lack this binding specificity for *E*. *coli*.

## Discussion

In this work, three independent alternatives have been designed and created in order to specifically detect the sentinel bacteria *E*. *coli* in drinking water samples. These three alternatives are based in an easy procedure dealing with biosensor chimera proteins, where a GFP amino-terminal domain is responsible for emitting fluorescence (after absorbance of UV light at 395 nm in a spectrophotometer cuvette) [44]. In contrast, the carboxyl-terminal domain at each chimera (hadrurin, pb5 or colS4 domains) is responsible for binding specifically to different structures in the external surface of *E*. *coli* cells (membrane, or the FhuA or OmpW proteins, respectively) [46–48]. Both domains (GFP and binding domains) are connected by a flexible artificial peptide linker (100 amino acids long), in order to facilitate free domains movements and to avoid steric complications between both at each chimera protein. We have carefully designed *in silico* these 100 amino acids linker in order to accomplish a totally flexible arm which is able to maintain the connectivity of both domains (GFP and binding domains) as a way to preserve their biological activities (fluorescence and binding, respectively). This total flexibility is shown in Figs 2, 4 and 5, and was secured by careful *in silico* testing for the absence of any α-helix nor β-sheet subsections, testing its whole sequence, as well as previous fragments and versions, for secondary structures [64].

The first biosensor chimera protein developed in this work, GFP-hadrurin, showed a very low binding to *E*. *coli* cells (to its outer membrane), independently of the sample processing (with or without filtering step used to get rid of free unbound chimera) [46,50]. The reason for this low labeling could be a low integration efficiency of the chimeric protein in the bacterial outer membrane. Another reason could be a steric inability of the biosensor chimera protein, as the small hadrurin α-helix carboxyl terminal domain could be blocked by surrounding linker and GFP domains. The reason for this is that the huge GFP amino terminal domain may partially encompass this domain, avoiding its access to the outer membrane, therefore causing low fluorescence labeling of bacterial cells.

In these and further experiments using confocal microscope, *E*. *coli* cells were always checked to test that they did not show autofluorescence under these experimental conditions, before using the corresponding samples with biosensor chimeras added (Fig 3G).

In a similar way, the experiments involving the second alternative, the GFP-pb5 chimera protein, showed a labile binding of this biosensor protein to *E*. *coli* cells, as adding a filtering step after the initial chimeric protein plus cell dilutions mix reduced significantly the obtained fluorescence labelling. This is in contrast to previous descriptions where free pb5 protein was able to bind to its FhuA bacterial receptor as a pure protein [55–58,65]. Purified pb5 is poorly soluble, as in published studies it precipitates at a concentration above 0.5 mg/mL or in the presence of imidazole [56]. Also, free pb5 protein tends to self-assembly, and these forms are not biologically active [58]. These described characteristics on free pb5 could be responsible for the low labelling detected in our experiments with the GFP-pb5 chimera protein, although in our experiments, no initial evidence of aggregation exists. Therefore, another possibility is that somehow the pb5 domain is suffering some conformational changes which makes its binding to the bacteria not stable enough to filter the solution before proceeding to the measure of fluorescence in the *E*. *coli* Analyzer. Further experiments with GFP-pb5 chimera were not carried out, as the biosensor performance was better with GFP-colS4 (see below).

However, the third chimeric protein designed in this work, GFP-colS4, showed excellent performance in our *in vitro* labelling tests for *E*. *coli*, by binding of its OmpW receptor in the outer membrane, as it has been described for this colicin [48,49].

Previous studies had shown the expression of colicin S4 in a wild type strain of *E*. *coli* bearing a plasmid which contained its gene [47]. In these experiments, colicin S4 was accumulated in the *E*. *coli* cytosol. However, the presence of OmpW receptors in this species made these cells sensitive to the action of this colicin S4, because a part of the cytosol expressed colicin was released into the culture medium in low concentrations, killing the bacteria [48]. Successful expression and purification of colicinS4 in *E*. *coli* by other authors forced the use of OmpW-mutant strains (as 5KΔmpW *E*. *coli* strain), or the simultaneous expression of the *csi* gene, responsible for immunity to colicin S4 *i*n *E*. *coli* [49,65].

However, in our expression system, these problems associated with the production of a biosensor chimera protein based on a colicin, as colicin S4, are absent. This is due to the fact that the bacterial factory used for expression of the fluorescent biosensor chimera protein is the actinomycete *S*. *albus*, which is not targeted by colicins.

In our experiments with the GFP-colS4 chimera protein, the fluorescent labelling of *E*. *coli* cells is good, even though colicin S4 domain theoretically would cause a translocation to the periplasm, through its OmpW receptor [49]. In the case of the chimera, however, probably its complete translocation has not taken place across the outer membrane, and part of it (at least the GFP domain) could stay on the outer side of the bacterial cell. The reason for this statement is that it has been described that versions of colicin S4 containing a His-tag are able to insert itself spontaneously in the outer membrane, but less frequently than wild type colicin S4 [48]. Therefore, this His-tag somehow hindered the insertion into the outer membrane, but not the binding to its receptor [48]. In our case, binding to OmpW seems to happen at high rates, and a nice fluorescence signal is also obtained, maybe indicating that the GFP domain has not been altered in its structure nor translocated across OmpW receptor (Fig 3). Although colicin S4 is known to bind to *E*. *coli* and finally to cause killing of these bacteria by forming pores in the cytoplasmic membrane, in our case, the short time for the experimental procedure and the fact that we actually see the *E*. *coli* cells under confocal microscope after labeling them with GFP-colS4 magic bullet, supports the idea that the eventual killing of these bacteria is not affecting our sensor approach. Moreover, our fast experimental design is not affected by the eventual killing of the bacterial cells after GFP-colS4 binding, since once the magic bullet is bound, we will get back the corresponding fluorescence from labelled cells.

OmpW expression in *E*. *coli* cells varies significantly in response to various environmental signals, such as temperature [65]. So, *E*. *coli* cells have shown greater resistance to colicin S4 when they grow at 23°C, compared to 37°C. The reason for this is a sharp reduction or even absence of OmpW receptor content at low temperatures as 23°C, as temperature regulates *ompW* gene expression at the transcriptional level [65]. This feature gives greater specificity to the method described in this study, as it would ensure that the *E*. *coli* contamination detected in the drinking water samples would be due to a recent faecal contamination (with high expression of OmpW protein as these cells were growing at 37°C), and not to a possible multiplication of the microorganism in the environment at low temperatures. Further studies in this sense will be carried out.

## Conclusions

Three different biosensor chimera proteins have been designed and tested for specific binding to *E*. *coli* cells in drinking water samples, in order to achieve an easy and fast detection method based on fluorescence. Each one of the designed biosensor chimera proteins contains a GFP domain placed at the amino terminus, in charge of emitting fluorescence once irradiated with UV light at 395 nm. Also, a magic bullet domain, for specific binding to some *E*. *coli* external structures (membrane, or FhuA and OmpW proteins) is present at the carboxyl terminus of each biosensor chimeric protein. Both important domains are linked through a 100 amino acids unfolded section, in order to ensure that no steric problems arise between them, and that the domain in charge of binding to the bacterial cell is freely moving with respect to the GFP domain. Selection of each one of the three binding domains (at the carboxyl-terminal of each chimera protein) has been carried out based on abundant literature on the binding specificities and requirements for each binding subunit: hadrurin is a simple and small lineal α-helix protein binding to the outer membranes of *Enterobacteriaceae* members; pb5 is part of the recognition structure of the central tail fibers near the base plate of T5 phage, in charge of initial binding to outer membrane iron ferrichrome transporter FhuA in *E*. *coli*, and colicin S4 is a bacteriocin with high specificity for *E*. *coli* outer membrane protein OmpW.

**Fig 7:**
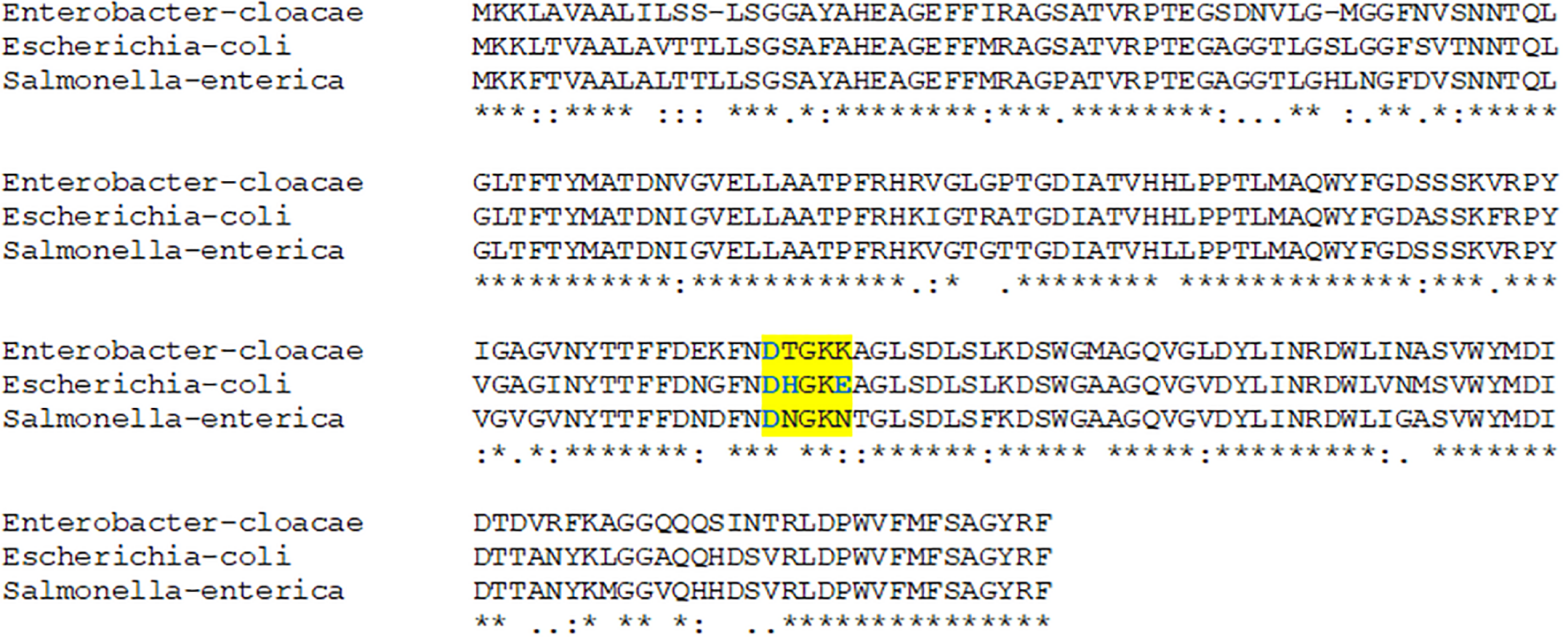
MUSCLE multiple sequence alignment of the OmpW proteins from *E*. *coli*, *S*. *enterica* var. *arizonae* and *E*. *cloacae*, highlighting in yellow the region encompasing the the Asp^116^, His^117^ and Glu^120^ amino acids of OmpW (in blue letters: D, H and E), which are freely exposed to the extracellular space and act as binding motifs for colicin S4 bacteriocin (as well as for GFP-colS4 chimera protein in this biosensor). As it is shown, the other two enterobacteria species do not have the conserved motif DH—E as in *E*. *coli*. Accesion numbers: SAI89619 (*E*. *cloacae*), BAA14788 (*E*. *coli*), OSE54138 (*S*. *enterica*).

Although some unspecific binding properties could arise due to the global structure of each one of the three chimera proteins, due to the presence of the amino-terminal GFP domain or to the central flexible synthetic peptide linker (showing a structure of a random coil lacking α-helixes nor β-sheets), other tested bacterial species as *S*. *enterica* and *E*. *cloacae* clearly ruled out this unspecificity, validating the initial experimental approach. Remarkably, these two other enterobacteria species do not have the conserved motif DHxxE as in E. coli (Fig 7). These three amino acids are the binding ones for colicin S4, as it has been described above [48].

The whole method relies also in the use of a specific *a la carte* portable detection device (*E*. *coli Analyzer*) which contains a LED source, a detection chamber, a photomultiplier and a converter of the fluorescence signal into mV data.

Two of these biosensor chimera proteins, the GFP-hadrurin and the GFP-pb5, showed a non-optimal performance with respect to binding parameters to *E*. *coli*, as the fluorescence signals obtained after the filtration step (necessary to get rid of unbound biosensor protein) were very low, probably due to some structural problems at the binding domains. However, the GFP-colS4 biosensor chimera protein showed nice stability and binding parameters, and was able to carry out the specific detection of *E*. *coli* with a linear range from 20 to 10^3^ CFU, and a lower detection level of 20 CFU in just 8 min, which are quite interesting features for its future development as a commercial detection method.

## Supporting information

S1 Table Detection signals obtained from 20 to 1000 CFUs for the three pathogens used in this study, using the *E*. *coli* Bioanalyzer device developed in the study. (PDF)
