# Supplementary material for: Development of a biosensor protein bullet as a fluorescent method for fast detection of Escherichia coli in drinking water
Source: PLoS One. 2018 Jan 5;13(1):e0184277. doi: 10.1371/journal.pone.0184277 (PMC5755745; doi:10.1371/journal.pone.0184277)
Supplement: S1 Table — (PDF) [file pone.0184277.s001.pdf]

CFU	20	50	180	360	500	1000
E. coli	256	298	381	460	486	527
	285	309	396	461	526	596
	264	287	418	427	472	488
Average	268,3	298,0	398,3	449,3	494,7	537,0
SD	8,6	6,4	10,7	11,2	16,2	31,6
S. enterica	260	261	255	267	251	251
	235	268	270	254	263	259
	269	256	248	244	259	268
Average	254,666667	261,666667	257,666667	255	257,666667	259,333333
SD	10,2	3,5	6,5	6,7	3,5	4,9
E. cloacae	241	266	255	255	281	235
	256	248	251	264	245	265
	235	231	242	266	256	261
Average	244	248,333333	249,333333	261,666667	260,666667	253,666667
SD	6,2	10,1	3,8	3,4	10,7	9,4
